# Digital ulcers in systemic sclerosis: their epidemiology, clinical characteristics, and associated clinical and economic burden

**DOI:** 10.1186/s13075-019-2080-y

**Published:** 2019-12-23

**Authors:** Kathleen Morrisroe, Wendy Stevens, Joanne Sahhar, Gene-Siew Ngian, Nava Ferdowsi, Catherine L. Hill, Janet Roddy, Jennifer Walker, Susanna Proudman, Mandana Nikpour

**Affiliations:** 1 Department of Medicine, The University of Melbourne at St. Vincent’s Hospital (Melbourne), 41 Victoria Parade, Fitzroy, Victoria 3065 Australia; 2 Department of Rheumatology, St Vincent’s Hospital (Melbourne), 41 Victoria Parade, Fitzroy, Victoria 3065 Australia; 3 Department of Medicine, Monash University, Clayton and Monash Health, 246 Clayton Road, Clayton, Victoria 3168 Australia; 4 Rheumatology Unit, Royal Adelaide Hospital, North Terrace, SA 5000 Australia; 5 Rheumatology Unit, The Queen Elizabeth Hospital, Woodville Road, Woodville, SA 5011 Australia; 6 Discipline of Medicine, University of Adelaide, Adelaide, SA 5000 Australia; 7 Department of Rheumatology, Royal Perth Hospital, Perth, Australia; 8 Rheumatology Unit, Flinders Medical Centre (Adelaide), Flinders Drive, Bedford Park, South Australia 5042 Australia

**Keywords:** Systemic sclerosis, Scleroderma, Digital ulceration, Economic burden

## Abstract

**Background:**

To determine the frequency and clinical characteristics of systemic sclerosis-related digital ulcers, and associated direct health care costs, quality of life, and survival.

**Methods:**

Digital ulcers (DUs) were defined as an area with a visually discernible depth and a loss of continuity of epithelial coverage. DU severity was calculated based on the physician reported highest number of new DUs at clinical review (mild = 1–5 DUs, moderate 6–10 DUs, severe > 10 DUs). Healthcare use was captured through data linkage, wherein SSc clinical data captured prospectively in a dedicated clinical database were linked with health services databases to capture hospital admissions, emergency department (ED) presentations and ambulatory care (MBS) utilization and cost for the period 2008–2015. Healthcare cost determinants were estimated using logistic regression.

**Results:**

Among 1085 SSc patients, 48.6% experienced a DU over a mean follow-up of 5.2 ± 2.5 years. Those who developed DUs were more likely to have diffuse disease subtype (34.9% vs 18.2%, *p* < 0.001), anti-Scl-70 antibody (18.9% vs 9.3%, *p* < 0.001), and a younger age at SSc onset (43.6 ± 13.9 vs 48.8 ± 14.0 years, *p* < 0.001) in addition to reduced health-related quality of life (HRQoL) measured by the SF-36 but without a significant impact on survival. SSc patients with a history of a DU utilized significantly more healthcare resources per annum than those without a DU, including hospitalizations, ED presentation, and ambulatory care services. Total healthcare services, excluding medications, were associated with an annual excess cost per DU patient of AUD$12,474 (8574-25,677), *p* < 0.001, driven by hospital admission and ED presentation costs.

**Conclusion:**

DUs place a large burden on the patient and healthcare system through reduced HRQoL and increased healthcare resource utilization and associated cost.

## Background

Systemic sclerosis (SSc) is an immune-mediated connective tissue disease characterized by vasculopathy and fibrosis of the skin and internal organs [1]. Although the pathogenesis of SSc remains unknown, autoimmune induced vascular injury is thought to be the cardinal event leading to structural and functional vasculature abnormalities resulting in fibroblast activation and consequent fibrotic changes characteristic of SSc [2, 3]. Important disease manifestations such as the Raynaud phenomenon (RP), digital ulceration, SSc renal crisis (SRC), and pulmonary arterial hypertension (PAH) are all thought to occur as a consequence of this autoimmune vasculopathy [4].

Digital ulcers (DUs) are a common and debilitating ischemic manifestation in SSc, representing end-organ damage from progressive vasculopathy and serving as a biomarker of disease severity and internal organ involvement [3, 5]. The presence of DU at any time is associated with DU recurrence, gastrointestinal (GIT) involvement, and increased SSc-related mortality [6, 7]. DUs are defined clinically as an area with a visually discernible depth and a loss of continuity of epithelial coverage, which can be denuded or covered by necrotic tissue and/or a scab [3]. DUs have a significant impact on the patient through the experience of extreme pain, global disability particularly of hand dysfunction, in addition to negatively impacting independence with activities of daily living, employment and work productivity [8]. Additionally, DUs require close contact with the healthcare system for meticulous wound care and sufficient analgesia, monitoring for potential complications such as the development of local and/or underlying bone infections that require prompt therapy. This therapy may be administered as an outpatient with oral antibiotics or may require hospitalization for intravenous antibiotics, prostanoid therapy, and/or consideration of surgical debridement or amputation. Furthermore, DU’s are associated with significantly poorer patient-reported health-related quality of life (HRQoL) [5].

Despite more than 50–70% of SSc patients reporting a history of DUs and 10% reporting a new DU within the prior 12 months [5, 9, 10], there is scant literature quantifying the burden of DUs in terms of healthcare utilization and associated economic cost. Therefore, we sought to evaluate the epidemiology, clinical characteristics, and outcomes (including HRQoL and survival) of DUs in our SSc cohort and to quantify associated healthcare resource utilization and direct cost through a data linkage study. We propose that such a comprehensive understanding of this burden would allow for the prioritization of future tailored research efforts and provide data to justify the provision of education and multidisciplinary healthcare services to reduce the risk of DU development and enable their prompt treatment.

## Methods

Consecutive SSc patients from four Australian states [Victoria (VIC), South Australia (SA), Western Australia (WA) and Tasmania (TAS)] prospectively enrolled in the Australian Scleroderma Cohort Study (ASCS), a multi-center study of risk and prognostic factors for clinically important outcomes in SSc, were included. The ASCS database annually collects comprehensive demographic and disease-related data. Written consent from all patients and ethical approval from all participating hospitals was obtained.

### Inclusion and exclusion criteria

We included all adult (> 18 years) SSc patients recruited in the ASCS between January 2008 (cohort inception) and December 2015 (when data linkage occurred). All patients fulfilled the American College of Rheumatology/European League Against Rheumatism Classification criteria for SSc [11].

### ASCS clinical data

SSc disease onset was defined as the first SSc clinical manifestation. Clinical manifestations and autoantibody status were defined as present if present ever from the time of SSc diagnosis. Autoantibodies are tested at enrollment. DU was defined clinically by the treating physician as an area on the digits with a visually discernible depth and a loss of continuity of epithelial coverage [3]. DU severity was calculated based on the physician reported highest number of new DUs on examination at clinical review with mild severity being defined as 1–5 new DUs, moderate by 6–10 new DUs and severe defined as > 10new DUs. GIT involvement included the presence of any of the following: gastro-esophageal reflux disease, reflux esophagitis, esophageal dysmotility, and/or esophageal stricture on endoscopy, intestinal dysmotility defined on barium and nuclear medicine studies, and diarrhea and/or fecal incontinence. Interstitial lung disease (ILD) was defined as present by characteristic fibrotic changes on high-resolution computed tomography (HRCT) lung [11]. Pulmonary arterial hypertension (PAH) was defined as present if diagnosed by right heart catheterization according to international criteria (defined as mean pulmonary arterial pressure (mPAP) of at least 25 mmHg and a pulmonary arterial wedge pressure (PAWP) of <= 15 mmHg) [12]. Detailed medication use data, prescribed at the discretion of the treating physician(s), are recorded at each visit for all patients allowing duration on therapy to be calculated. Patient status (alive or dead) was censored in January 2016.

### HRQoL

HRQoL was recorded annually using the Medical Outcome Short Form-36 (SF-36), a validated instrument for measuring HRQoL in SSc [13]. A score between 0 and 100 is calculated which is standardized to normative population HRQoL scores. Scores below 50 indicate worse HRQoL than the background population with one standard deviation represented by 10 points. These scores can be summarized into the physical component score (PCS) and mental component score (MCS) as was done in this study using the average patient PCS and MCS score from enrollment to the last follow-up.

### Data linkage

The ASCS database of de-identified SSc patients’ demographic and disease-related data was merged with hospital, emergency department (ED) and ambulatory care (Medicare Benefits Schedule (MBS)) databases, through the Australian Institute of Health and Welfare (AIHW), thereby capturing all healthcare use. All data were stored and analyzed within a remote-access secure computing environment (Secure Unified Research Environment (SURE)).

### Healthcare utilization and costing methodology

Hospital admission and ED presentation databases contain information on the primary diagnosis, in addition to the Diagnosis Related Grouping (DRG), Urgency Related Grouping (URG), and an associated weighted unit used to estimate the financial cost. The cost was calculated based on the financial year of admission and the corresponding weighted value. The MBS lists the ambulatory care services for which a government-funded payment can be claimed [14]. MBS cost was calculated using the fee that the government covers for all Australians (the total “benefit payable fee”). Medication cost was determined from the PBS Dispensed Price for Maximum Quantity (DPMQ) paid for the standard dose of each medication, which is the cost the government contributes towards each medication dispensed.

### Statistical analysis

Data are presented as mean ± standard deviation (SD) for normally distributed and median (25th–75th) for non-normally distributed continuous variables, and as number (percentage) for categorical variables. Differences in frequency were tested using chi-square and Fisher’s exact tests. Univariable and multivariable logistic regression in addition to ordinal regression for DU severity were used to determine the associations of DU with healthcare utilization and cost. Kaplan-Meier (K-M) survival curves were used to estimate survival in patients with and without DU and by severity. To estimate HRQoL, the patients’ PCS and MCS median scores from enrollment to the last follow-up were calculated and served as a threshold for defining high and low HRQoL in those with and without DU. Variables with a *p* value < 0.05 in univariable regression or variables deemed to be of clinical significance to the outcome with a *p* value < 0.20 were included in the multivariable logistic regression analysis.

A two-tailed *p* value of 0.05 or less was considered statistically significant. All statistical analyses were performed using STATA 14.0 (StataCorp LP, College Station, TX, USA).

## Results

### Patient characteristics

Our cohort consisted of 1085 SSc patients, of whom 527 (48.6%) had experienced a history of a DU over a mean follow-up of 5.2 ± 2.5 years. SSc patients with a DU history compared with those without a history of DU were younger at SSc onset (43.6 ± 13.9 vs 48.8 ± 14.0 years, *p* < 0.001) and had longer disease duration (12.2 ± 10.5 vs 10.1 ± 9.9 years, *p* = 0.001). Furthermore, they were more likely to be of male gender (18.0% vs 11.5%, *p* = 0.002) and have diffuse disease subtype (dcSSc) (34.9% vs 18.2%, *p* < 0001). At censorship, there were fewer SSc patients alive with a history of DU than without a history of DU (78.1% vs 83.1%, *p* = 0.05). In terms of autoantibody profile, those with a history of DU were more likely to be positive for either antitopoisomerase-1 (Scl-70) antibody or anti-RNA Polymerase (RNAP) III (18.9% vs 9.3%, *p* < 0.001 and 16.9% vs 11.8%, *p* = 0.05, respectively) than SSc patients without a history of a DU. Moreover, those with DU were more likely to have telangiectasia, calcinosis, joint contractures, GIT involvement, and SSc-related cardiopulmonary manifestations (namely PAH and ILD) in addition to the co-morbidity of peripheral vascular disease (PVD) (Table 1). Furthermore, those with a history of a DU were more likely to be treated with vasodilator therapies such as calcium channel blockers (CCB) and iloprost than those without DUs in addition to certain endothelial receptor antagonists (ERAs) and phosphodiesterase-5 inhibitors (PDE5) which were prescribed as a PAH specific therapy in those with PAH. The presence of DUs was associated with reduced HRQoL reflected by the PCS of the SF-36 (36.1 ± 10.4 vs 39.2 ± 11.2, *p* < 0.001) (Table 1).

**Table 1:** Characteristics of SSc patients by DU status^

Patient characteristics (*n* = 1085)	DU	No DU	*p* value
	Mean ± SD or *n*(%)	Mean ± SD or *n*(%)	
Number of patients	527 (48.6%)	558 (51.4%)	
Demographics			
Age on SSc onset*, years	43.6 ± 13.9	48.8 ± 14.0	< 0.001
Disease duration at recruitment, years	12.2 ± 10.5	10.1 ± 9.9	0.001
Gender female	432 (81.9%)	494 (88.5%)	0.002
Male	95 (18.0%)	64 (11.5%)	0.002
Disease subtype limited disease subtype	342 (65.%)	455 (81.8%)	< 0.001
Diffuse disease subtype	184 (34.9%	101 (18.2%)	< 0.001
Caucasian ethnicity	476 (95.9%)	502 (93.7%)	0.09
Follow-up, years	5.2 ± 2.5	4.7 ± 2.4	0.001
Alive at censorship	363 (78.1%)	412 (83.1%)	0.05
Autoantibody profile**			
Anti-centromere pattern ANA (*n* = 1059)	213 (41.2%)	268 (49.5%)	0.007
Scl 70 + ve (*n* = 1046)	96 (18.9%)	50 (9.3%)	< 0.001
RNA polymerase III + ve (*n* = 693)	57 (16.9%)	42 (11.8%)	0,05
Clinical manifestations***			
Telangiectasia ever	504 (95.6%)	452 (81.0%)	< 0.001
Calcinosis ever	299 (56.7%)	179 (32.1%)	< 0.001
Joint contractures	321 (60.9%)	153 (27.4%)	< 0.001
GIT involvement	478 (90.7%)	456 (81.7%)	< 0.001
SSc renal crisis	19 (3.6%)	14 (2.5%)	0.293
PAH^#^	87 (16.5%)	65 (11.7%)	0.02
ILD	185 (53.1%)	149 (26.7%)	0.003
Co-morbidities			
CVA	33 (6.3%)	30 (5.4%)	0.53
Diabetes mellitus	37 (7.0%)	52 (9.3%)	0.17
PVD	26 (4.9%)	6 (1.1%)	< 0.001
Smoking history (current or ever)	275 (52.2%)	275 (52.2%)	0.19
Medications			
Calcium channel blocker (CCB)	414 (78.6%)	311 (55.7%)	< 0.001
PDE5 inhibitor			
Sildenafil	99 (18.8%)	46 (8.2%)	< 0.001
Tadalafil	11 (2.1%)	10 (1.8%)	0.724
Endothelial receptor antagonist (ERA)			
Ambrisentan	30 (5.7%)	18 (3.2%)	0.05
Bosentan	100 (18.9%)	81 (14.5%)	0.05
Macitentan	17 (3.2%)	13 (2.3%)	0.37
Iloprost	148 (28.1%)	10 (1.8%)	< 0.001
Topical vasodilator	71 (13.5%)	10 (1.8%)	< 0.001
HRQoL			
Physical component score (PCS)	36.1 ± 10.4	39.2 ± 11.2	< 0.001
Mental component score (MCS)	46.3 ± 12.8	46.1 ± 13.9	0.84

*Abbreviations*: *DU* digital ulceration, *PAH* pulmonary arterial hypertension, *ILD* interstitial lung disease, *GIT* gastrointestinal tract, *CVA* cerebrovascular accident, *PVD* peripheral vascular disease, *ACA* anticentromere, *Scl-70* antitopoisomerase-1, *RNAP* anti-RNA polymerase III, *CCB* calcium channel blockers, *ERAs* endothelial receptor antagonists, *PDE5* phosphodiesterase-5 inhibitors

Health-related quality of life (HRQoL), was defined using the SF-36 study short form which provides a score range from 0 to 100.·Scores below 50 indicate worse HRQoL than the population normative score and every 10 points indicates 1 standard deviation.·These scores can be summarized into the physical component score (PCS) and mental component score (MCS), scores below 50 indicate worse HRQoL than the population normative score and every 10 points indicates 1 standard deviation

^DU status defined as the physicians reported presence of any history of DU

*SSc onset defined as the first symptom of SSc (Raynaud phenomenon or other) *disease duration defined as from first non-Raynaud’s disease manifestation

**n denotes the number of patients who underwent the bloods test thereby determining if they were positive or negative

***clinical manifestations defined as present if ever present from SSc diagnosis

^#^PAH diagnosed on right heart catheterization (RHC) according to international criteria [11]

### Patient characteristics by DU severity

In our cohort, 75.6% of patients had mild DU, 14.6% had moderate DU and 9.9% had severe DU (Table 2). Increasing DU severity was associated with male gender (*p* = 0.01), dcSSc (*p* < 0.001) and the presence of Scl-70 antibody (*p* < 0.001), while the presence of ACA was associated with less severe DUs (*p* < 0.001). In terms of clinical manifestations, increasing DU severity was associated with the presence of joint contractures (*p* < 0.001) and ILD (*p* < 0.001). In terms of DU complications in the 12 months preceding their clinical review, increasing DU severity was associated with more frequent hospitalizations for DU management, in addition to increased use of intravenous (IV) antibiotics and IV prostanoid therapy (*p* = 0.04, *p* = 0.02, and *p* = 0.03 respectively) (Table 2). Furthermore, increasing DU severity was associated with progressively worsening HRQoL reflected by the PCS of the SF-36 (*p* = 0.02) (Table 2).

**Table 2:** Patient characteristics by DU severity*

Patient characteristics by number of DU	Mild DU*	Moderate DU*	Severe DU*	*p* value
	*n* (%) or mean ± SD Median (IQR 25th–75th)	*n* (%) or mean ± SD Median (IQR 25th–75th)	*n* (%) or mean ± SD Median (IQR 25th–75th)	
Patient number	308 (74.8%)	60 (14.6%)	44 (10·7%)	
Demographics				
Age at SSc onset, years	43.9 ± 14.5	42.8 ± 12.1	43.4 ± 12.3	0.56
Female gender	286 (84.9%)	54 (83.1%)	29 (65.9%)	0.01
Limited disease subtype	230 (68.5%)	30 (46.2%)	12 (27.3%)	< 0.001
Alive	239 (79.9%)	51 (85.0%)	28 (68.3%)	0.12
Smoke (past or current)	169 (50.2%)	33 (50.7%)	28 (63.6%)	0.24
DU severity (highest no of DU)				
Mild (1–5 digital ulcers on exam)	337 (75.6%)	65 (14.6%)	44 (9.9%)	
Moderate (6–10 digital ulcers on exam)				N/A
Severe (> 10 digital ulcers on exam)				
Autoantibody profile**				
Anti-centromere pattern ANA (*n* = 437)	159 (48.2%)	17 (26.6%)	5 (11.6%)	< 0.001
Scl 70 + ve (*n* = 432)	53 (16.3%)	19 (30.2%)	21 (48.8%)	< 0.001
RNA polymerase III + ve (*n* = 300)	35 (15.4%)	17 (36.9%)	4 (15.4%)	0.03
Clinical manifestations***				
Telangiectasia ever	321 (95.3%)	64 (98.5%)	44 (100%)	0.18
Calcinosis ever	188 (55.8%)	42 (64.6%)	29 (65.9%)	0.53
Joint contractures	194 (57.6%)	55 (84.6%)	39 (88.6%)	< 0.001
GIT involvement	305 (90.5%)	62 (95.4%)	39 (88.6%)	0.38
SSc Renal Crisis	12 (3.6%)	1 (1.5%)	3 (6.8%)	0.35
PAH^#^	59 (17.5%)	6 (9.2%)	5 (11.4%)	0.17
ILD	104 (30.9%)	25 (38.5%)	27 (61.4%)	< 0.001
Hospitalized in the last 12 months for				
Digital ulcers	71 (23.1%)	22 (36.7%)	17 (38.6%)	0.04
Intravenous antibiotics therapy	34 (11.0%)	13 (21.7%)	11 (25.0%)	0.02
Intravenous prostanoids	60 (19.5%)	18 (30.0%)	16 (36.4%)	0.04
Surgical debridement	22 (7.1%)	6 (10.0%)	5 (11.4%)	0.18
Medications				
CCB	258 (76.6%)	54 (83.1%)	38 (86.4%)	0.21
PDE5 inhibitor	64 (18.9%)	17 (26.2%)	13 (29.5%)	0.05
ERAs	72 (21.4%)	9 (13.8%)	8 (18.8%)	0.36
Iloprost	92 (27.3%)	22 (38.9%)	21 (47.7%)	0.02
Topical vasodilators	44 (13.1%)	10 (15.4%)	9 (20.5%)	0.39
HRQoL				
Physical component score (PCS)	36.6 ± 10.6	35.3 ± 9.6	31.3 ± 9.8	0.02
Mental component score (MCS)	46.5 ± 13.3	43.4 ± 12.6	45.9 ± 12.4	0.38

*Abbreviations*: *DU* digital ulceration, *PAH* pulmonary arterial hypertension, *ILD* interstitial lung disease, *GIT* gastrointestinal tract, *CVA* cerebrovascular accident, *PVD* peripheral vascular disease, *ACA* anticentromere, *Scl-70* antitopoisomerase-1, *RNAP* anti-RNA Polymerase III, *CCB* calcium channel blockers, *ERAs* endothelial receptor antagonists, *PDE5* phosphodiesterase-5 inhibitors, *SSc* scleroderma

Health-related quality of life (HRQoL) was defined using the SF-36 study short form which provides a score range from 0 to 100.·Scores below 50 indicate worse HRQoL than the population normative score and every 10 points indicates 1 standard deviation.·These scores can be summarized into the physical component score (PCS) and mental component score (MCS), scores below 50 indicate worse HRQoL than the population normative score and every 10 points indicates 1 standard deviation

*DU severity was calculated based on the physician reported highest number of new DUs on examination at clinical review (mild 1–5 new DU, moderate 6–10, and severe > 10 new DU)

### Survival analysis in those with and without DU

**Fig. 1:**
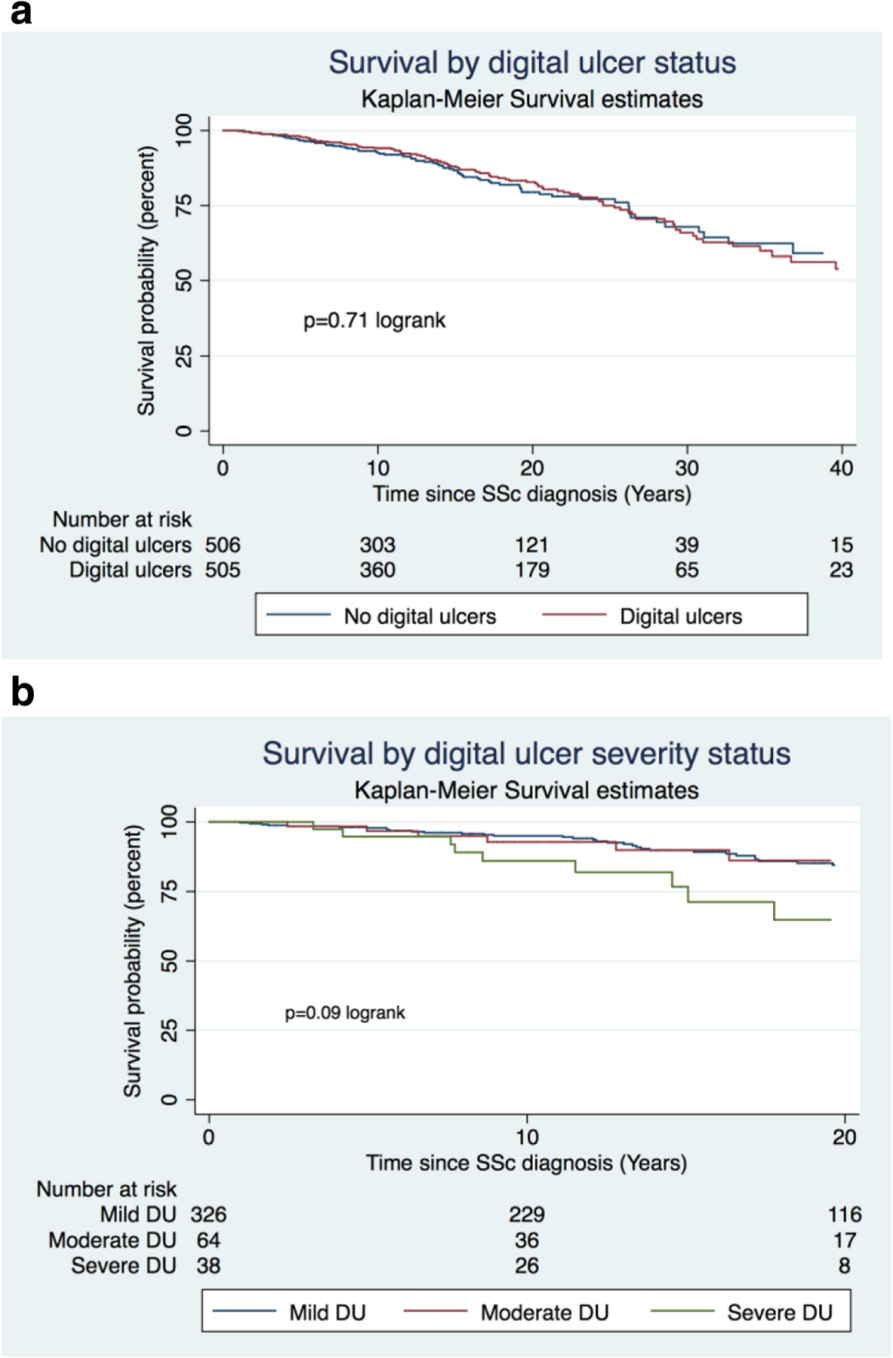
**a** Kaplan-Meier survival curves by DU status. **b** Kaplan-Meier survival curves by DU severity

There was no significant difference in survival between those with and without DU in our SSc cohort (Fig. 1a). The mean time to death from SSc onset was 15.8 ± 12.8 years for those with DU and 17.4 ± 10.8 years for those without DU, *p* = 0.11. Classifying by DU severity, those with severe DU had a shorter time from SSc onset to death than those with moderate or mild DU (15.5 ± 10.5 years, 15.2 ± 9.9 years, 17.0 ± 11.1 years, *p* = 0.09 respectively), albeit not statistically significant (Fig. 1b).

### Healthcare utilization and associated direct cost

#### Hospital admission and cost

During 2008–2015, SSc patients with a history of DU had a higher annual frequency of hospital admissions than those without a history of DU (2.1 (1–3.7) vs 1.5 (1–2.8), *p* < 0.001) and had a longer average length of stay (LOS) while in hospital (2.3 (1.2–4.3) vs 1.8 (1.1–3.9) days, *p* < 0.001) (Table 3). The most common reasons for admission in those with DUs was related to their SSc, followed by chronic ulceration and Raynaud phenomenon, while for those without DUs, pneumonia followed by a complication of their SSc and lower respiratory tract infections (LRTI) were the most common admission diagnoses (Table 3). During this same time period, the cost associated with hospitalization was significantly higher for those with DUs than those without DUs with a total average hospital cost per patient of AUD$23,888 (7413–71,434) vs AUD$13,535 (1846–43,797), *p* < 0.001) and an annual admission cost per patient of AUD$4107 (2654–6621) for those with DUs compared with AUD$3439 (2172–5374) for those without DUs, *p* = 0.001 (Table 4). Hospital costs did not differ significantly with increasing DU severity, with an annual admission cost per patient with mild DU of AUD$4232 (2716–6843), moderate DU of AUD$4297 (2963-6207), and severe DU of AUD$4614 (2889–6978), *p* = 0.98 (Table 5).

**Table 3:** Healthcare utilization in SSc by DU status between 2008 and 2015

Characteristics per patient	DU *n* (%) or mean ± SD, median (IQR 25th–75th) (*n* = 527)	No DU *n* (%) or mean ± SD, median (IQR 25th–75th) (*n* = 558)	*p* value
Hospitalization			
% of patients admitted to hospital (2008–2015)	466 (88.4%)	454 (81.4%)	< 0.001
Average annual hospital admissions per patient	2.1 (1–3.7)	1·5 (1–2.8)	< 0.001
Average LOS per patient per admission	2.3 (1.2–4.3)	1·8 (1.1–3.9)	< 0.001
Reason 3 reasons for admission			
1	Systemic sclerosis	Pneumonia	
2	Chronic ulcer	Systemic sclerosis	
3	Raynaud’s syndrome	LRTI	
ED			
% of patients presenting to ED (2008–2015)	375 (71.2%)	356 (63.8%)	0.01
Average annual ED presentations per patient	2 (1–4)	2 (1–3)	0.001
Top 3 reasons for ED presentation			
1	Chest pain	Chest pain	
2	Lobar pneumonia	Acute LRTI	
3·	Dyspnea	Abdominal pain	
MBS			
% of patients utilizing an MBS service (2008–2015)	522 (99.1%)	556 (99.6%)	0.23
Average annual MBS services utilized per patient	58 (39–91)	55 (35–86)	0.03
Top 3 MBS services utilized			
1	Pathology (44.9%)	Pathology (42.9%)	
2	Professional (29.9%)	Professional (32.9%)	
3	Allied Health Service (14.7%)	Allied Health Service (11.2%)	

*Abbreviations*: *DU* digital ulceration, *MBS* medicare benefits schedule, *LOS* length of stay, *ED* emergency department, *PVD* peripheral vascular disease, *LRTI* lower respiratory tract infection

**Table 4:** The financial cost (AUD$) associated with healthcare utilization in SSc by DU status

Characteristics per patient	DU Mean ± SD, median (IQR 25th–75th) AUD$	No DU Mean ± SD, median (IQR 25th–75th) AUD$	*p* value
Total healthcare cost*			
Total cost per patient (2008–2015)	46,364 (24,561–92,582)	33,890 (15,987–66,905)	< 0.001
Median annual cost per patient	7854 (5596–11,404)	7060 (4968–9893)	0.001
Hospitalization cost			
Total admission cost per patient (2008–2015)	23,888 (7413–71,434)	13,535 (1846–43,797)	< 0.001
Median annual admission cost per patient	4107 (2654–6621)	3439 (2172–5374)	0.001
ED presentation			
Total ED cost per patient (2008–2015)	822 (0–2137)	449 (0–1594)	0.001
Median ED cost per patient	449 (0–872)	421 (0–720)	0.001
MBS			
Total MBS cost per patient (2008–2015)	16,839 (10,302–25,689)	16,395 (9786–24,619)	0.34
Median annual MBS cost per patient	2509 (1621–3786)	2444 (1511–3770)	0.25
Total Medication cost			
Total medication cost per patient (2008–2015)	1273 (662–14,609)	1126 (553–17,940)	0.79

*Abbreviations*: *DU* digital ulceration, *MBS* medicare benefits schedule, *ED* emergency department, *CCB* calcium channel blockers, *ERAs* endothelial receptor antagonists, *PDE5* Sphosphodiesterase-5 inhibitors (PDE5)

*Medication cost not included in total healthcare cost

**Table 5:** The financial cost of healthcare utilization in SSc by DU severity*

Healthcare cost	Mild DU*	Moderate DU*	Severe DU*	*p* value
	Mean ± SD, median (25th–75th) or *n*(%)	Mean ± SD, median (25th–75th) or *n*(%)	Mean ± SD, median (25th–75th) or *n*(%)	
Total healthcare cost				
Total cost/pt. (2008–2015)	45,933 (24,823–99,604)	48,199 (30,472–79,802)	79,827 (36,861–117,822)	0.44
Median annual cost/pt.	8269 (5784–11,713)	7881 (6220–11,433)	9048 (5429–10,700)	0.46
Hospital cost				
Total cost/pt. (2008–2015)	25,317 (7039–74,211)	33,783 (7573–58,526)	51,567 (15,752–98,245)	0.29
Median annual cost/ pt	4232 (2716–6843)	4297 (2963–6207)	4614 (2889–6978)	0.98
ED cost				
Total cost/pt. (2008–2015)	800 (0–2082)	843 (0–2526)	966 (422–2965)	0.46
Median annual cost/pt.	449 (0–871)	449 (0–898)	610 (409–968)	0.36
MBS cost				
Total cost/pt. (2008–2015)	17,017 (10,395–26,756)	16,280 (9639–26,534)	14,077 (8268–21,193)	0.35
Median annual cost/pt.	2635 (1676–4000)	2347 (1644-3935)	2330 (1578–2968)	0.12
Medication cost				
Total cost/pt. (2008–2015)	1189 (646–8541)	1395 (732–6007)	1239 (928–7683)	0.45

*Abbreviations*: *DU* digital ulceration, *MBS* medicare benefits schedule, *ED* emergency department, *CCB* calcium channel blockers, *ERAs* endothelial receptor antagonists, PDE5 phosphodiesterase-5 inhibitors

*DU severity was calculated based on the physician reported highest number of new DUs on examination at clinical review (mild 1–5 new DU, moderate 6–10, and severe > 10 new DU)

#### ED presentation and cost

The frequency of ED presentations during 2008–2015 was higher among SSc patients with a history of DU than those without a DU (71.2% vs 63.8%, *p* = 0.01) with an annual average ED presentations frequency per DU patient of 2 (1–4) compared with 2 (1–3) for those without DUs, *p* = 0.001 (Table 3). The most common reasons for ED presentation in those with DUs were chest pain, followed by lobar pneumonia and dyspnoea, while for those without a history of DU, chest pain, acute LRTI, and abdominal pain were the three most frequent reasons for ED presentation (Table 3). The cost associated with ED presentations during the period of 2008–2015 was higher for those with DUs than those without DUs (total cost/pt. AUD$822 (0–2137) vs AUD$449 (0–1594), *p* = 0.001), with an annual ED cost per DU patient of AUD$449 (0–872) vs AUD$421 (0–720) for those without DUs, *p* = 0.001 (Table 4). Similar to hospital cost, DU severity did not significantly alter the healthcare cost associated with ED presentations, with an annual average ED cost per patient with mild DU of AUD$449 (0–871), moderate DUs of AUD$449 (0–898), and severe DUs of AUD$610 (409–968) respectively, *p* = 0.36 (Table 5).

#### MBS utilization and cost

Almost all patients within our cohort utilized a MBS service during 2008–2015 (99.1% with DU and 99.6% without DUs, *p* = 0.23) with an annual average per patient service utilization of 58 (39–91) for those with DUs and 55 (35–86) for those without DUs, *p* = 0.03. The most commonly utilized MBS services were similar in both those with and without DUs and included pathology, professional visits, and allied health visits (Table 3).

Furthermore, the most commonly utilized allied health services by those with DUs in descending order were wound care, psychology, and podiatry while in those without DUs psychology, podiatry, and physiotherapy were the most commonly utilized services. The healthcare costs associated with MBS service utilization during the period of 2008–2015 was not significantly higher for those with and without DUs (total MBS cost per patient AUD$16,839 (10,302–25,689) vs AUD$16,395 (9786–24,619), *p* = 0.343), with an annual MBS cost per DU patient of AUD$2509 (1621–3786) vs AUD$2444 (1511–3770) for those without DUs, *p* = 0.26 (Table 4). As seen with hospital and ED costs, MBS costs did not differ with DU severity, with an annual MBS cost per patient with mild DU of AUD$2635 (1676–4000), moderate DU of AUD$2347 (1644–3935), and severe DU of AUD$2330 (1578–2968) respectively, *p* = 0.12 (Table 5).

#### Medication utilization and associated cost

Vasodilator therapies, their duration of use, and associated cost were assessed by DU status including the use of CCB and iloprost in addition to certain endothelial receptor antagonists (ERAs) and phosphodiesterase-5 inhibitors (PDE5) which were prescribed as a PAH specific therapy for those who had a concurrent diagnosis of PAH. The average medication cost per patient between 2008 and 2015 was not significantly different for those with and without a DU (AUD$1273 (662–14,609) vs AUD$1126 (553–17,940), *p* = 0.79) (Table 4). Furthermore, increasing DU severity was not associated with a significantly higher medication cost (AUD$1189 (646–8541) for mild DU, AUD$1395 (732–6007) for moderate DU and AUD$1239 (928–7683) for severe DU, *p* = 0.45) (Table 5). Medication cost by patient number, DU status, and specific therapy is further summarized in Additional file 1: Table S1.

#### Total healthcare utilization and associated cost

The total healthcare cost (including hospital, ED, and MBS cost) for our cohort during 2008–2015 was significantly higher among those with DU compared to those without DUs with a total average healthcare cost per patient of AUD$46,364 (24,561–92,582) vs AUD$33,890 (15,987–66,905), *p* < 0.001) and an annual cost per patient of AUD$7854 (5596–11,404) for those with DUs compared with AUD$7060 (4968–9893) for those without DUs, *p* = 0.001 (Table 4). Total healthcare cost did not increase significantly with DU severity, with an annual healthcare cost per patient with mild DU of AUD$8269 (5784–11,713), moderate DU of AUD$7881 (6220–11,433), and severe DU of AUD$9048 (5429–10,700) respectively, *p* = 0.46 (Table 5).

Determinants of above-median total annual healthcare cost (and its components) associated with DUs by univariable logistic regression are summarized in Additional file 1: Table S2. By multivariable logistic regression, determinants of total annual healthcare cost in those with DUs included increasing age at SSc onset (OR 1.03, *p* < 0.001), the presence of PAH (OR 1.8, *p* = 0.05), and the use of Iloprost (OR 1.8, *p* = 0.02) (Table 6). Determinants of each component of this healthcare cost, including hospitalizations, ED presentations, and MBS utilization, in DUs were also assessed in multivariable logistic regression and are summarized in Table 6.

**Table 6:** Determinants of above-median annual total healthcare cost and its components in SSc-DU in multivariable logistic regression

	OR (95%CI)	*p* value
Determinants of annual total healthcare cost		
Female	1.21 (0.7–2.1)	0.48
Age at SSc onset*, years	1.03 (1.0–1.1)	< 0.001
Caucasian ethnicity	0.55 (0.2–1.6)	0.27
Diffuse subtype	0.71 (0.4–1.2)	0.19
ILD	1.38 (0.9–2.2)	0.16
PAH^#^	1.78 (0.9–3.2)	0.05
PDE-5-inhibitor	1.0 (0.6–1.8)	0.99
Iloprost	1.77 (1.1–2.8)	0.02
Determinants of hospital cost		
Female	1.20 (0.7–2.1)	0.49
Caucasian ethnicity	0.83 (0.3–2.3)	0.72
Age at SSc onset*, years	1.03 (1.0–1.1)	0.001
Diffuse subtype	0.97 (0.6–1.6)	0.91
PAH^#^	1.32 (0.7–2.3)	0.35
ILD	1.27 (0.8–1.9)	0.29
Iloprost	1.59 (1.0–2.5)	0.04
PDE5 inhibitor	1.13 (0.6–1.9)	0.68
Determinants of ED cost		
Female	1.29 (0.7–2.3)	0.39
Caucasian ethnicity	0.96 (0.3–2.8)	0.94
Age at SSc onset*, years	1.02 (0.9–1.0)	0.06
Diffuse subtype	0.79 (0.5–1.3)	0.37
PAH^#^	2.89 (1.4–5.9)	0.004
ILD	1.29 (0.8–2.1)	0.33
Determinants of MBS cost		
Female	1.79 (1.0–3.2)	0.05
Age at SSc onset*, years	1.02 (1.0–1.1)	0.001
Caucasian ethnicity	0.82 (0.3–2.5)	0.001
Diffuse subtype	0.68 (0.4–1.1)	0.10
PAH^#^	2.41 (1.3–4.5)	0.001
DU severity**		
Mild	Baseline	
Moderate	1.05 (0.6–1.9)	0.88
Severe	0.71 (0.3–1.6)	0.39
CVA	3.35 (1.1–10.4)	0.04
Diabetes mellitus	1.51 (0.6–3.8)	0.39

*Abbreviations*: *DU* digital ulceration, *PAH* pulmonary arterial hypertension, *ILD* interstitial lung disease, *CVA* cerebrovascular accident, *CCB* calcium channel blockers, *ERAs* endothelial receptor antagonists, *PDE5* phosphodiesterase-5 inhibitors

*SSc onset defined as the first symptom of SSc (Raynaud phenomenon or other) *disease duration defined as from first non-Raynaud’s disease manifestation

**DU severity was calculated based on the physician reported highest number of new DUs on examination at clinical review (mild 1–5 new DU, moderate 6–10, and severe > 10 new DU)

^#^PAH diagnosed on right heart catheterization (RHC) according to international criteria [11]

Hospital cost was associated with increasing age at SSc onset (OR 1.03, *p* < 0.001) and joint contractures (OR 2.1, *p* = 0.001), while ED cost was associated with the presence of PAH (OR 2.9, *p* = 0.01) and MBS cost was associated with female gender (OR 1.8, *p* = 0.05), increasing age at SSc onset (OR 1.0, *p* = 0.001), Caucasian ethnicity (OR 0.8, *p* = 0.001), the presence of PAH (OR 2.4, *p* = 0.001), and CVA (OR 3.4, *p* = 0.04).

## Discussion

Our study is the first data linkage study describing the epidemiology, clinical characteristics, and outcomes of DUs in a large SSc cohort in addition to comprehensively quantifying their healthcare utilization and associated economic burden. In our cohort of 1085 SSc patients, 48.6% of SSc patients experienced a DU over a mean follow-up of 5.2 ± 2.5 years. Consistent with the literature [9, 15, 16], those who developed DUs, compared with those who did not, were more likely to have dcSSc (34.9% vs 18.2%, *p* < 0.001), be positive for Scl-70 (18.9% vs 9.3%, *p* < 0.001), and have a younger age at SSc onset (43.6 ± 13.9 vs 48.8 ± 14.0 years, *p* < 0.001). Additionally, SSc patients who developed DUs, compared with those who did not, were more likely to have PAH (16.5% vs 11.7%, *p* = 0.02) and more likely to have ILD (53.1% vs 26.7%, *p* = 0.003) [17, 18]. SSc patients with a history of a DU utilized significantly more healthcare resources on an annual basis than those without a DU, including hospitalizations, ED presentation, and ambulatory care services. Total healthcare services, excluding medication cost, were associated with an excess cost per DU patient of AUD$12,474 (8574–25,677), *p* < 0.001, driven by hospital admission and ED presentation costs. There was no difference in ambulatory care cost in those with and without DUs, which may be related to our study not including the costs of public hospital outpatient clinics including wound care, podiatry, and hand therapy. Younger age at SSc onset and the presence of PAH were the main determinants of overall healthcare cost in SSc patients with a history of DU. Despite DU being associated with morbidity and reduced HRQoL, our study indicates that their presence alone does not reduce survival.

Consistent with the literature [19, 20], we have shown that SSc patients with a history of DUs utilize more healthcare resources than those without DUs, particularly a higher frequency of hospital admissions with a higher average LOS. In a retrospective UK study of 1168 SSc patients [19], 17.4% of their cohort experienced severe digital vasculopathy with 16.6% experiencing a DU over an 18 month period. Of these, 12.1% were admitted to hospital for IV prostacyclin, 1.5% required parenteral, and 4.8% received oral antibiotics. Consistent with our data, they found that the frequency of hospital admissions among patients with digital vasculopathy was higher compared with those without digital vasculopathy (37.9% vs 6.6%, *p* < 0.001). Similarly, in an observational cohort of 189 SSc patients with incident DU [20], 23% developed a DU-related complication including gangrene, auto-amputation, and/or infections requiring systemic antibiotic therapy in 36 patients and 58.7% of patients required more than one hospitalization with 67% having a LOS of more than 1 day. In the only study to estimate healthcare cost associated with DUs, an Italian pilot study of 20 SSc patients estimated a mean annual patient cost from a healthcare service perspective of €23,730 ± 11,409 [21]. This cost was inclusive of hospitalization, day procedure, and medication cost, in addition to general practitioner and specialist appointment costs and was driven by the cost of iloprost infusions in Italy (€34,693 for 6 cycles of infusions) [21].

Although increasing DU severity in our study was associated with increasing frequency of hospitalizations in the 12 months preceding the clinical review for inpatient management of DUs (*p* = 0.04), increasing use of IV antibiotic therapy (*p* = 0.02) and increasing use of IV prostanoids (*p* = 0.04), increasing DU severity was not associated with an increased overall total healthcare cost (*p* = 0.44). The reason behind this discrepancy between increasing DU severity and overall healthcare cost is unclear, but may be due to the lack of standard DU severity classification criteria or the inability of rheumatologists to reliably grade DUs [5]. This further highlights the need for a standard and reliable DU categorization that enables the treating physician to determine those patients who are likely to experience increased DU disease burden and thus need frequent monitoring and/or complex management.

Despite the presence of DU treatment recommendations, a large proportion of patients do not receive DU-specific medications [5]. In our cohort, 78.6% of DU patients were treated with a CCB, 28.1% received at least one course of iloprost infusion, and 13.5% received topical vasodilator therapy. In Australia, government-subsidized treatment with an ERA or a PDE-5-inhibitor is only available for SSc-PAH patients, which would explain the low use of these medications in our cohort and sole use in those with concurrent PAH (with 20.8% of DU patients having been treated with a PDE-5-inhibitor and 27.9% an ERA). In an ideal world, SSc patients with DUs should be managed by a dedicated multidisciplinary team compromising a patient educator, wound care nurse, hand therapist, podiatrist, and rheumatologist and perhaps even a hand and vascular surgeon. Patient education is crucial to effective and efficient DU management including the importance of smoking cessation, keeping warm in cold weather, hand hygiene, and meticulous wound care in addition to seeking early medical advice when ulcers appear [5]. Given the high prevalence of local skin infections complicating DUs, such a clinic would provide the opportunity for an early medical review and prescription of antibiotics in addition to appropriate and sufficient analgesia. Furthermore, an assessment can be made for early hospitalization if there is an inadequate response to treatment, thus preventing potential systemic complications such as osteomyelitis, improving HRQoL and survival, and thereby reducing the economic cost as all care would be provided in the one visit and location.

In regard to quality of life, our cohort’s HRQoL data echoes those of other studies [5, 20] showing that the presence of DUs has a significant impact on HRQoL compared with those without DUs in a disease whose overall HRQoL is reported to be worse than most other chronic diseases including heart failure and diabetes [22]. Furthermore, our study has shown for the first time that HRQoL deteriorates further with increasing DU severity, particularly highlighted in the PCS (PCS 36.6 ± 10.6. in mild DU, 35.3 ± 9.6 moderate DU and 31.3 ± 9.8 for those with severe DU, *p* = 0.02). Improving HRQoL in SSc patients is a real area of unmet need, which requires a more targeted understanding before improvements can be made.

Strengths of our study include its well-characterized SSc cohort followed prospectively over a substantial period of time in addition to its data linkage methodology ensuring the reliable ascertainment of healthcare utilization and economic burden. Limitations include the inability to differentiate between different types, locations and time to healing of digital ulcers such as fingertip ulcers and proximal interphalangeal ulcers nor describe the specific surgical interventions used such as sympathectomy or botox, in addition to the potential for underestimating the true cost as hospitalizations in the private hospital sector were not included nor were the cost of public outpatient clinics or allied health services not captured by the MBS. The lack of inclusion of cost related to public outpatient clinics including wound care, podiatry, and hand therapy is likely significant given complex SSc patients are often managed through tertiary referral public hospitals and their affiliated outpatient clinics. Unfortunately, these costs cannot be obtained using data linkage methodology as they are hospital-specific costs and not billed through the MBS. Furthermore, some patients may not have accessed healthcare services for their minor DUs and our study did not estimate the impact of DUs on employment and work productivity; these are important aspects to consider when estimating the true financial cost of DUs.

## Conclusions

DUs are a serious complication of SSc and place a large burden on the healthcare system and the patient through reduced HRQoL, incremental healthcare resource utilization and associated cost without impacting survival. To reduce the clinical burden of DUs, additional research is needed to determine effective interventions and management plans such as the development of a DU specific multidisciplinary clinic.

## Supplementary information

**Additional file 1.** **Table S1.** Medication cost by DU status. **Table S2.** Determinants of above median annual total healthcare cost in SSc-DU in univariate logistic regression.

## Data Availability

Data is available upon request to the corresponding author.

## Abbreviations

Scleroderma, SSc: Systemic sclerosis
lcSSc: Limited cutaneous systemic sclerosis
dcSSc: Diffuse cutaneous systemic sclerosis
DU: Digital ulceration
ILD: Interstitial lung disease
PAH: Pulmonary arterial hypertension
ASCS: Australian Scleroderma Cohort Study
ACR/EULAR: American College of Rheumatology/European League Against Rheumatism
GIT: Gastrointestinal tract
ACA: Anti-centromere
Scl-70: Anti-topoisomerase-1
RNAP III: Anti-RNA polymerase
mRSS: Modified Rodnan Skin Score
RP: Raynaud’s phenomenon
ED: Emergency department
LOS: Length of stay
MBS: Medicare Benefit Schedule
CVA: Cerebrovascular accident
PVD: Peripheral vascular disease
CCB: Calcium channel blockers
ERAs: Endothelial receptor antagonists
PDE5: Phosphodiesterase-5 inhibitors
HRQoL: Health-related quality of life
