# Supplementary material for: Digital ulcers in systemic sclerosis: their epidemiology, clinical characteristics, and associated clinical and economic burden
Source: Arthritis Res Ther. 2019 Dec 23;21:299. doi: 10.1186/s13075-019-2080-y (PMC6929369; doi:10.1186/s13075-019-2080-y)
Supplement: Supplementary file 1 — Additional file 1. Table S1. Medication cost by DU status. Table S2. Determinants of above median annual total healthcare cost in SSc-DU in univariate logistic regression. [file 13075_2019_2080_MOESM1_ESM.docx]

Additional file 1

**Table S1.** Medication cost by DU status

| **Medication** | **DU**  **n**  **Median (IQR 25^th^-75^th^)** | **No DU**  **n**  **Median (IQR 25^th^-75^th^)** | **p-value** |
| --- | --- | --- | --- |
| Total median medication cost/pt (2008-2015) | 1,273 (662-14,609) | 1,126 (553-17,940) | 0·79 |
| CCB  Patient number  Median cost/pt (2008-2015) | 290  808 (454-1,239) | 207  738 (450-1,249) | 0·55 |
| PDE5 inhibitor  Sildenafil  Patient number  Median cost/pt (2008-2015)  Tadalafil  Patient number  Median cost/pt (2008-2015) | 55  1,737 (892-3,189)  6  9,440 (7,924-18,123) | 25  2,660 (901-3,186)  9  14,278 (9,728-17,940) | 0·77  0·50 |
| ERAs  Bosentan  Patient number  Median cost/pt (2008-2015)  Macitentan  Patient number  Median cost/pt (2008-2015)  Ambrisentan  Patient number  Median cost/pt (2008-2015) | 67  63,885 (37,710-99,097)  7  36,309 (31,603-49,469)  21  96,422 (66,481-109,306) | 51  70,776 (33,722-112,777)  5  34,292 (34,196-34,292)  10  52,610 (33,487-96,781) | 0·43  0·56  0·17 |
| Iloprost  Patient number  Median cost/pt (2008-2015) | 148  2,383 (1,254-4,427) | 10  1,851 (973-3,793) | 0·05 |

Abbreviations: digital ulceration (DU), calcium channel blockers (CCB), endothelial receptor antagonists (ERAs), phosphodiesterase-5 inhibitors (PDE5)

**Table S2.** Determinants of above median annual total healthcare cost in SSc-DU in univariate logistic regression

| **Type of cost** | **Total healthcare cost** | | **Hospital cost** | | **ED cost** | | **MBS cost** | |
| --- | --- | --- | --- | --- | --- | --- | --- | --- |
| **Patient characteristic**  **(n=527)** | **OR (95%CI)** | **p-value** | **OR (95%CI)** | **p-value** | **OR (95%CI)** | **p-value** | **OR (95%CI)** | **p-value** |
| Demographics  Female  Age at onset of SSc*, years  Caucasian ethnicity  Diffuse disease | 1·03 (0·6-1·7)  1·03 (1·0-1·1)  0·62 (0·2-1·6)  1·13 (0·8-1·7) | 0·91  <0·001  0·33  0·52 | 0·97 (0·6-1·6)  1·02 (1·0-1·1)  0·79 (0·3-2·0)  1·26 (0·9-1·8) | 0·91  0·002  0·62  0·23 | 1·38 (0·8-2·3)  1·01 (0·9-1·0)  1·14 (0·4-3·2)  0·81 (0·5-1·3) | 0·25 0·09  0·81  0·35 | 1·65 (1·1-2·6)  1·03 (1·0-1·1)  0·71 (0·3-1·1)  0·64 (0·4-0·9) | 0·03  <0·001  0·46  0·01 |
| Clinical manifestations**  Telangiectasia ever  Calcinosis ever  Joint contractures  GI involvement  Renal Crisis  PAH^#^  ILD | 1·11 (0·5-2·7)  1·26 (0·9-1·8)  1·94 (1·3-2·8)  1·12 (0·6-2·2)  1·59 (0·6-4·2)  2·26 (1·4-3·7)  0·39 (0·9-2·0) | 0·82  0·22  0·001  0·74  0·34  0·001  0·08 | 1·66 (0·7-4·1)  1·17 (0·8-1·7)  2·01 (1·4-2·9)  1·26 (0·7-2·5)  -  1·76 (1·1-2·9)  1·39 (0·9-2·0) | 0·27  0·39  <0·001  0·49  -  0·02  0·08 | 1·05 (0·4-2·9)  1·42 (0·9-2·2)  1·32 (0·9-2·1)  1·68 (0·8-3·6)  1·05 (0·4-3·1)  2·35 (1·2-4·5)  1·33 (0·8-2·1) | 0·92  0·12  0·21  0·18  0·93  0·01  0·22 | 1·02 (0·4-2·4)  1·24 (0·9-1·8)  0·98 (0·7-1·4)  1·41 (0·8-2·6)  1·09 (0·4-2·7)  2·13 (1·3-3·5)  1·26 (0·9-1·8) | 0·96  0·23  0·88  0·26  0·86  0·002  0·20 |
| DU Severity***  Mild DU  Moderate DU  Severe DU | baseline  0·94 (0·5-1·7)  1·14 (0·6-2·2) | 0·82  0·71 | baseline  1·26 (0·7-2·3)  1·22 (0·6-2·4) | 0·45  0·56 | baseline  1·21 (0·6-2·5)  1·58 (0·7-3·6) | 0·61  0·29 | baseline  0·79 (0·5-1·4)  0·54 (0·3-1·0) | 0·43  0·06 |
| Co-morbidities  CVA  Diabetes Mellitus  PVD | 1·42 (0·7-2·9)  2·43 (1·2-5·1)  1·94 (0·8-4·7) | 0·36  0·02  0·14 | 0·71 (0·3-1·5)  -  1·94 (0·8-4·7) | 0·36  -  0·14 | 1·08 (0·5-2·6)  0·95 (0·4-2·0)  2·23 (0·7-6·7) | 0·87  0·89  0·16 | 2·77 (1·3-6·1)  2·83 (1·3-5·9)  1·60 (0·7-3·6) | 0·01  0·01  0·26 |
| Medications  CCB  PDE5 inhibitor  ERAs  Iloprost  Topical vasodilators | 0·97 (0·6-1·5)  1·64 (1·1-2·6)  1·38 (0·9-2·1)  1·73 (1·2-2·6)  1·00 (0·6-1·7) | 0·91  0·04  0·15  0·01  1·0 | 0·83 (0·5-1·3)  1·55 (0·9-2·5)  1·31 (0·9-2·0)  1·58 (1·1-2·4)  1·17 (0·7-2·0) | 0·42  0·06  0·22  0·02  0·58 | 0·88 (0·5-1·5)  1·29 (0·8-2·2)  1·33 (0·8-2·3)  1·45 (0·9-2·3)  0·85 (0·5-1·6) | 0·61  0·36  0·29  0·13  0·62 | 1·13 (0·7-1·7)  1·21 (0·8-1·9)  1·59 (1·1-2·4)  1·05 (0·7-2·4)  1·39 (0·8-2·3) | 0·57  0·39  0·03  0·82  0·19 |

Abbreviations: digital ulceration (DU), pulmonary arterial hypertension (PAH), interstitial lung disease (ILD), cerebrovascular accident (CVA), calcium channel blockers (CCB), endothelial receptor antagonists (ERAs), phosphodiesterase-5 inhibitors (PDE5)

*SSc onset defined as the first symptom of SSc (Raynaud phenomenon or other) *disease duration defined as from first non-Raynaud’s disease manifestation

** clinical manifestations defined as present if ever present from SSc diagnosis

*** DU severity was calculated based on the physician reported highest number of new DUs on examination at clinical review (mild 1-5 new DU, moderate 6-10 and severe >10new DU)

^#^PAH diagnosed on right heart catheterization (RHC) according to international criteria [11]
